# Progress and Challenges in Tackling Child and Adolescent Obesity

**DOI:** 10.34763/devperiodmed.20172103.177178

**Published:** 2017-10-28

**Authors:** Marie-Laure Frelut

**Affiliations:** 1 The European Childhood Obesity Group, Albi, France

The childhood obesity epidemic and its early deleterious consequences were an unexpected phenomenon in a world where technical progress was supposed to improve health status rather than disrupt a balance acquired over generations.

The analysis of epidemiological data published in 195 countries showed that the prevalence of obesity almost doubled between 1980 and 2015, nowadays reaching 108 million children all over the world. Although this prevalence is lower in children than in adults, its rate of increase is steeper. This consortium confirmed that more than two thirds of deaths linked to high BMI are due to cardiovascular diseases [1]. When European prevalence data on childhood obesity became available, a Northern-South gradient was evidenced: prevalence in Mediterranean countries is almost twice as high as in most Northern ones. Steeper increases were noticed in countries facing rapid changes in levels of life and lifestyles [2]. Contrary to all expectations, countries that produced fruit and vegetables and had developed the traditional Mediterranean diet were facing the worst conditions.

These contradictions gave birth to passionate debates and myriads of studies. The state of the art shifted within 20 years from a focus on the way energy balance could be disrupted and restored to an open debate, where many different fields, such as biology, medicine, surgery, psychology, anthropology, pharmacology, economics and law were investigated. Although therapeutic consequences are still limited, scientific knowledge and the fight between prevention and treatment has turned into an agreement on both: their respective necessity and further development, leading to warning about treatment safety.

Childhood obesity offered some of the best opportunities of medical and scientific progress. For example, taking into account the field of appetite regulation: in 1999, three years after J Friedman had discovered that leptine deficient mice were extremely obese and did not develop secondary sexual characters [3], Farooqi et al identified consanguineous families with similar leptin, a cytokine inhibiting appetite, or leptin receptor abnormalities and successfully treated children by human recombinant leptin [4]. A gene hunting phase was started but it quickly became obvious that major mutations were seldom the cause and that human gene polymorphism explained under 10 % of the individual susceptibility to obesity. Later on, however, Wabistch et al showed that leptin and leptin receptor mutations may not be as rare as initially thought, provided sensitive techniques of detection are used [5]. Brain-gut-adipose tissue interactions through many neuro-hormonal pathways, which were unknown two decades ago, are now promising fields of investigation. New concepts, such as epigenetics, also faced a tremendous development, bridging the gap between Darwin’s and Lamarck’s concepts of the way character could be inherited: the molecular environment within cells modulates the way gene expression takes place or is inhibited, nutrition being a major contributor to the intracellular milieu interieur. The consequences of these variations are transmitted from the father or mother to children without any mutation, which explains why the grandchildren of women who had faced famine are at a higher risk of developing obesity, type 2 diabetes or cardiovascular diseases [6]. Bioinformatics has in the meantime allowed the discovery that gut microbiota contains about 10 times more bacteria than the whole body number of cells, leading to suspect it plays a significant metabolic role. Indeed, the gut flora of obese mice transferred to axenic mice induces weight and fat mass gain without increase in energy intake. Several teams are now trying to decipher the role of the transmission of this microbiota within the family and especially between obese mothers and their newborns and its role in order to prevent and treat obesity or avoid relapse occurring after bariatric surgery. Let us add to this picture that we now know adipose tissue is not any more simply white or brown but also pink and beige with shifts from one category to another and is a genuine resizable endocrine organ storing lipids but also producing many hormones and cytokines.

This vast amount of fundamental discoveries has brought both a huge advantage and disadvantage to paediatricians: the identification of early risk factors put them in the front line but has not yet provided caregivers that face the challenge of obesity treatment with very efficient solutions. That is why being a good clinician, able to detect the components of each child’s obesity pattern, is still extremely important. Let us take a quick example: obese children are usually reluctant to move and spent a lot of time facing screens. School performance then decreases and the kid may react with sugar craving, being susceptible and impulsive. In such a case, sleep should be investigated and craving or binge eating differentiated from overeating because of a decreased feeling of satiation [7]. Sleep apnoeas are major contributors to abnormal behaviours that require specific investigation and treatment. Childhood obesity is also a consequence of circadian rhythm disruption. Another common problem, which is, however, difficult to solve, is the reduction in physical activity. Obese children often complain of muscular and bone pains that limit their capability and willingness to move. A careful clinical examination, the contribution of a physiotherapist, or a physical activity specialist is useful to understand how far the position of the centre of gravity and postural control are modified by obesity. A psychologist’s intervention may be necessary in order to overcome the shame of being obese and shifting from virtual activity and power gained on video games to real life. Physical activity should, therefore, be adapted and part of a comprehensive management strategy. This is why the European Childhood Obesity Group recently issued a review of current knowledge about physical activity in obese children and adoelscents [8]. A multidisciplinary knowledge and approach are necessary and paediatric teams dealing with child and adolescent obesity should be organized from that perspective.

Since obesity treatment is challenging and hardly successful, prevention remains a key issue. For it to go beyond the step of incantations, social, psychological and economic mechanisms that underlie its development have to be taken into account and several non-medical specialists should be involved in order to launch a genuine Public Health action, as required by the World Health Organization. The role of lawyers is also essential in order to frame the legal protection at the national and European levels that children and adolescents deserve in a world dominated by economics. Such prevention policies, in which the parents have key roles to play, has allowed stabilization in several European countries [1].
